# Time-Restricted Ketogenic Diet in Huntington's Disease: A Case Study

**DOI:** 10.3389/fnbeh.2022.931636

**Published:** 2022-07-29

**Authors:** Matthew C. L. Phillips, Eileen J. McManus, Martijn Brinkhuis, Beatriz Romero-Ferrando

**Affiliations:** ^1^Department of Neurology, Waikato Hospital, Hamilton, New Zealand; ^2^Mental Health Services for Older People, Tauranga Hospital, Tauranga, New Zealand

**Keywords:** Huntington's disease, neurodegeneration, energy metabolism, mitochondria dysfunction, metabolic strategy, fasting, ketogenic diet

## Abstract

Huntington's disease (HD) is a progressive, fatal neurodegenerative disorder with limited treatment options. Substantial evidence implicates mitochondria dysfunction in brain and skeletal muscle in the pathogenesis of HD. Metabolic strategies, such as fasting and ketogenic diets, theoretically enhance brain and muscle metabolism and mitochondria function, which may improve the clinical symptoms of HD. We report the case of a 41-year-old man with progressive, deteriorating HD who pursued a time-restricted ketogenic diet (TRKD) for 48 weeks. Improvements were measured in his motor symptoms (52% improvement from baseline), activities of daily living (28% improvement), composite Unified HD Rating Scale (cUHDRS) score (20% improvement), HD-related behavior problems (apathy, disorientation, anger, and irritability improved by 50–100%), and mood-related quality of life (25% improvement). Cognition did not improve. Weight remained stable and there were no significant adverse effects. This case study is unique in that a patient with progressive, deteriorating HD was managed with a TRKD, with subsequent improvements in his motor symptoms, activities of daily living, cUHDRS score, most major HD-related behavior problems, and quality of life. Our patient remains dedicated to his TRKD, which continues to provide benefit for him and his family.

## Introduction

Huntington's disease (HD) is a progressive, fatal neurodegenerative disorder that afflicts 10.6–13.7 out of every 100,000 people in the west (McColgan and Tabrizi, 2018). Clinically, HD is characterized by involuntary movements, cognitive decline, and behavior problems such as apathy, irritability, and depression. Striking neurodegenerative changes occur in the striatum (Reiner et al., 1988), the primary input region of the basal ganglia, which acts as a central “hub” in a series of cortico-subcortical circuits involved in the appropriate organization and execution of movement, cognition, and behavior (O'Callaghan et al., 2014). Neurodegeneration in HD is linked to a CAG trinucleotide repeat expansion in the huntingtin gene on chromosome 4, which codes for a mutant huntingtin protein that aggregates to form intranuclear inclusion bodies within neurons (Ross and Tabrizi, 2011). The mutant huntingtin protein induces neuron dysfunction by disrupting multiple mechanisms related to transcription, translation, proteostasis, axonal transport, and mitochondria function (Ross and Tabrizi, 2011; McColgan and Tabrizi, 2018). Treatment for HD is largely supportive and new strategies are needed.

Substantial evidence implicates mitochondria dysfunction as a key player in the pathogenesis of HD (Kim et al., 2010; Oliveira, 2010). Impaired striatal glucose metabolism occurs years before the motor symptoms occur (Grafton et al., 1992; Antonini et al., 1996). Expression of peroxisome proliferator-activated receptor-γ coactivator-1α (PGC-1α), a major regulator of mitochondria biogenesis and energy metabolism, is decreased (Cui et al., 2006; Kim et al., 2010). Morphologically, neuron mitochondria are reduced in number and fragmented (Kim et al., 2010). Functionally, postmortem studies show moderate to severe defects in respiratory chain complexes II, III, and IV (Gu et al., 1996; Browne et al., 1997). Beyond the brain, reduced PGC-1α expression and mitochondria dysfunction also occur in peripheral tissues, especially skeletal muscle (Chaturvedi et al., 2009; Ciammola et al., 2011). The progressive decline in mitochondria function leads to a chronic cell energy shortage which cripples metabolically active cells, particularly neurons and muscle cells.

Metabolic strategies, such as fasting and ketogenic diets, enhance brain and muscle metabolism and mitochondria function (Phillips, 2022), which could theoretically mitigate the clinical symptoms of HD. Fasting is a voluntary abstinence from food and drink that permits water, calorie-free fluids, or limited calorie-restricted meals for specified periods of time (typically, at least 12 h in humans) (Longo et al., 2021). Ketogenic diets are high-fat, adequate-protein, low-carbohydrate diets in which carbohydrates are restricted to <50 g daily (O'Neill, 2020). Both strategies stimulate the body to produce ketones, which can be used by neurons and muscle cells as an energy source. During a typical western diet, the concentration of the primary blood ketone, beta-hydroxybutyrate (BHB), rarely exceeds 0.5 mmol/L, whereas fasting and ketogenic diets can induce a state of physiological ketosis during which its concentration exceeds 0.5–0.6 mmol/L (VanItallie and Nufert, 2003). Compared with glucose, BHB provides more energy per unit oxygen due to an increase in the free energy of ATP hydrolysis and the supply of TCA cycle intermediates (Veech et al., 2001). Importantly, both fasting and ketogenic diets also upregulate PGC-1α, mitogenesis, and mitophagy (Miller et al., 2018; de Cabo and Mattson, 2019), renewing the mitochondria pool. Through these mechanisms, metabolic strategies might be capable of suppressing the HD process—for example, compared with mice on a normal diet, the HD pathophysiological and clinical phenotype is mitigated in mice fasted every other day (Duan et al., 2003). However, to our knowledge, neither fasting nor a ketogenic diet have ever been applied to a patient with HD.

## Case Study

We report the case of a 41-year-old male former resource center assistant of northern European background who presented to our HD clinic with clinically and genetically confirmed HD. He had been diagnosed in a different clinic 4 years previously after developing several months of mild choreiform movements on a background of several years of worsening anxiety and impaired social functioning. Gene testing had revealed 47 CAG repeats. At the time of our review, he was deteriorating in his motor symptoms, cognition, and daily function and required assistance from family members using cutlery, self-grooming, and getting dressed. He also had significant behavior problems characterized by frequent, prolonged episodes of apathy, anger, and irritability. The apathy was so severe he required prompting to eat and would rarely ingest enough food, resulting in 5–6 kg of weight loss over the preceding 12 months. The behavior problems were undermining his relationship with his fiancée, who was also his caregiver. Medical history was significant for type 1 diabetes, which was diagnosed 3 years previously (1 year after the HD diagnosis) and controlled with insulin glusiline 5 units tds and insulin glargine 9 units daily. His diabetes specialist routinely documented a mean blood glucose level of 9–11 mmol/L and his control was erratic, with hypoglycemic episodes (<3 mmol/L) occurring on most days despite multiple insulin adjustments. He had recently trialed olanzapine for his anger and irritability, but this worsened his blood glucose control and made him feel sedated, so it was ceased. Due to this negative experience as well as concerns over further antipsychotics aggravating his diabetes, our patient was reluctant to trial any other drugs. Family history was significant for HD in his paternal grandfather (confirmed on autopsy), father (42 CAG repeats), and only brother (42 CAG repeats). Socially, he lived with his fiancée and spent most of his time at home, no longer able to work due to his symptoms. Neurological examination revealed marked chorea affecting the face, neck, trunk, and limbs. The limbs additionally exhibited pronounced dystonic posturing during walking, leading to occasional stumbling. Our patient was 178 cm in height and 62.7 kg in weight, resulting in a calculated body-mass index of 19.8 kg/m^2^. MRI brain revealed atrophy of the basal ganglia consistent with HD. His fiancée and mother were concerned about his ability to remain at home and were exploring care facility options.

**Figure 1:**
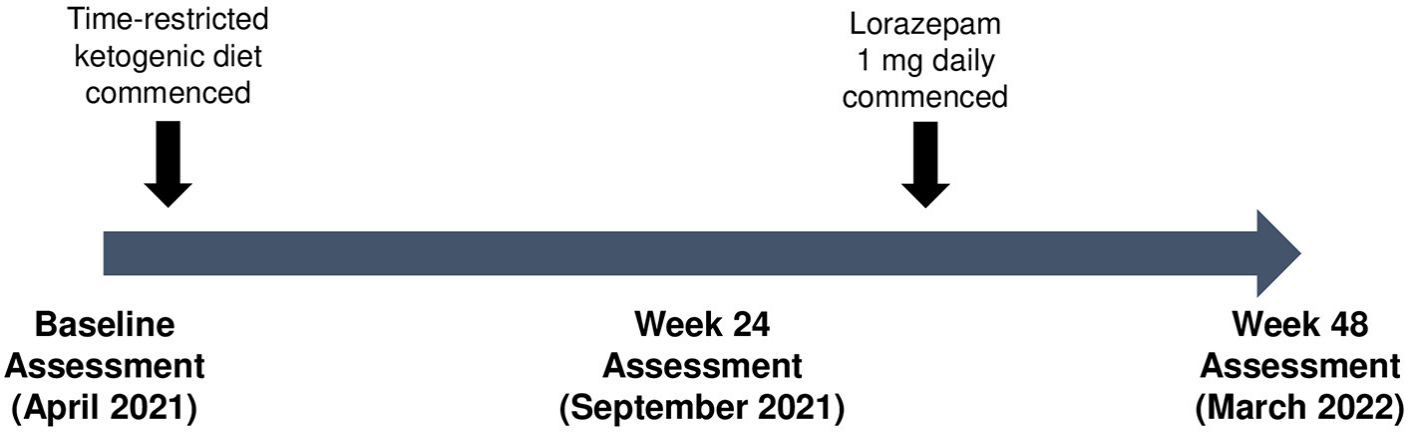
Patient timeline.

Given the progressive deterioration in HD symptoms, a 48-week “combined” metabolic strategy consisting of a time-restricted ketogenic diet (TRKD) was offered (Figure 1). The time-restriction component of the TRKD involved reducing feeding times from three meals a day, plus snacks, to two meals a day, with no snacks. Our patient chose the timing of the two meals every day and up to 1 h was allowed for each meal. The modified ketogenic diet, which consisted largely of whole foods, was 60% fat, 30% protein, 5% fiber, and 5% net carbohydrate by weight and consisted mainly of green vegetables, meats, eggs, nuts, seeds, creams, and natural oils. After obtaining written informed consent we provided a booklet containing guidelines, recipes, and space to record daily (bedtime) blood glucose and ketone levels, which were measured with a blood glucose and ketone monitor (CareSens Dual, Pharmaco Diabetes, Auckland, New Zealand). The lead investigator provided regular email contact as needed. There were no other lifestyle alterations during the TRKD.

We performed a 2-h clinical assessment during the week prior to commencing the TRKD, followed by assessments during weeks 24 and 48. Clinical assessments evaluated motor symptoms, cognition, daily function, HD-related behavior problems, and quality of life. The clinical assessments were always performed at the same hour of the day and by the same treatment-blinded assessor. Motor symptoms were assessed by a neurologist using the Unified HD Rating Scale (UHDRS^®^) Total Motor Score (TMS) (scores range from 0 to 124, with lower numbers indicating improved motor symptoms) (Hungtington Study Group, 1996). Cognition was measured by a neuropsychologist using the Symbol Digit Modalities Test (SDMT) (scores range from 0 to 110, with higher numbers indicating improved cognition) and the Stroop Word Reading and Color Tests (no maximum score range, with higher numbers indicating improved cognition) (Stroop, 1935). Daily function was measured by a neurologist using the UHDRS^®^ Total Functional Capacity (TFC) score (scores range from 0 to 13, with higher numbers indicating improved function) (Hungtington Study Group, 1996) and the caregiver-reported HD Activities of Daily Living (HD-ADL) scale (scores range from 0 to 51, with lower numbers indicating improved function) (Bylsma et al., 1993). A composite measure of motor symptoms, cognition, and daily function was calculated using the composite UHDRS (cUHDRS) score (calculated using the TMS, SDMT, Stroop Word Reading Test, and TFC, with higher numbers indicating improved composite outcomes) (Schobel et al., 2017). Behavior problems were measured by a neuropsychologist using the Problem-Behaviors Assessment—short form (PBA-s) questionnaire (scores range from 0 to 16 for each of 11 behavior problems, with lower numbers indicating improved behavior) (Craufurd et al., 2001). Quality of life was measured using the caregiver-reported HD health-related Quality of Life (HDQoL) questionnaire (scores range from 0 to 100 across four domains of physical function, cognition, mood-self, and worries, with higher numbers indicating improved quality of life) (Ho et al., 2019). Body weight and blood markers were measured at each assessment. An adverse effects questionnaire was administered to the patient and caregiver at weeks 24 and 48.

**Figure 2:**
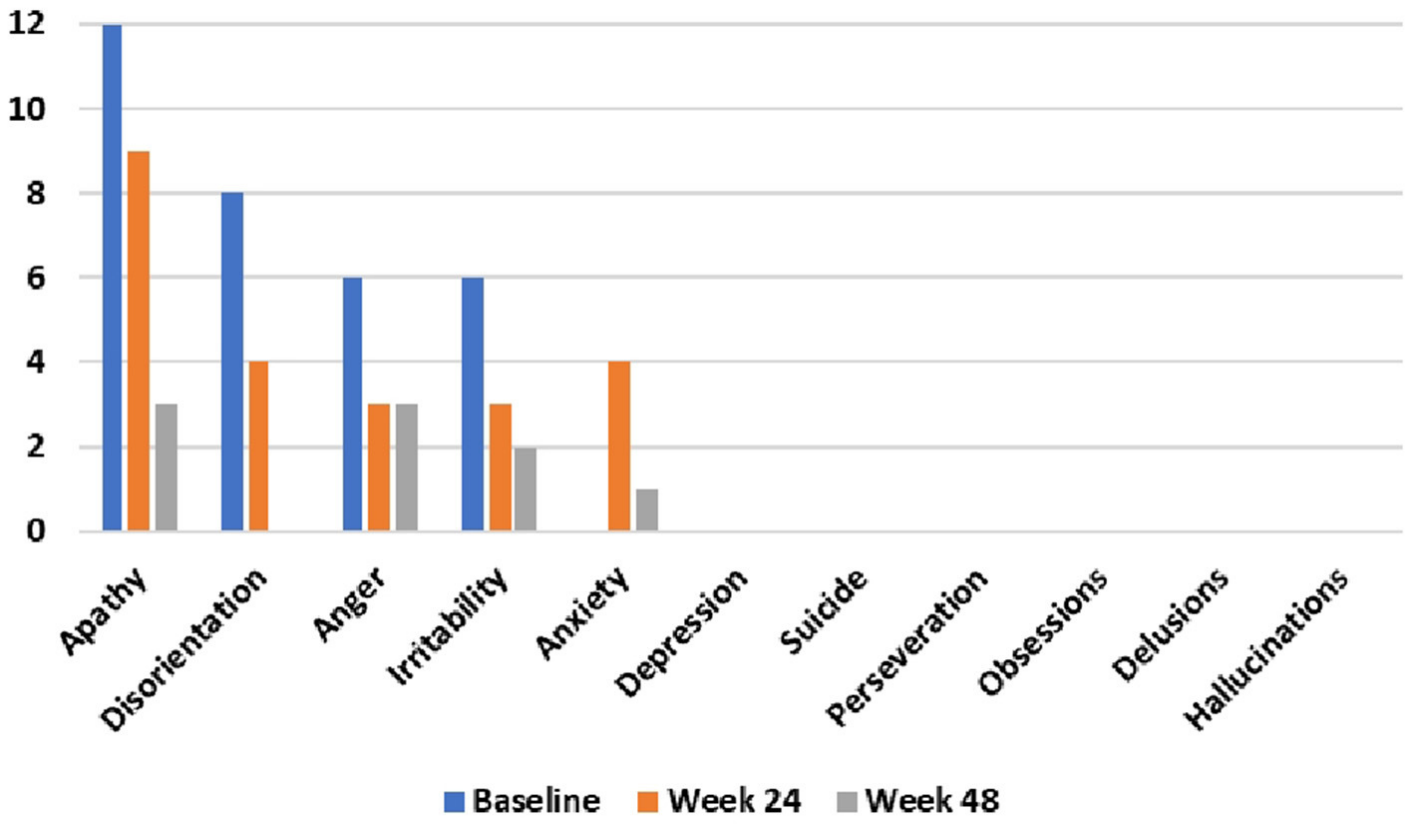
Problem-behaviors assessment—short form (PBA-s) outcomes at baseline, 24 weeks, and 48 weeks after commencing the time-restricted ketogenic diet (lower numbers indicate improved behavior).

**Figure 3:**
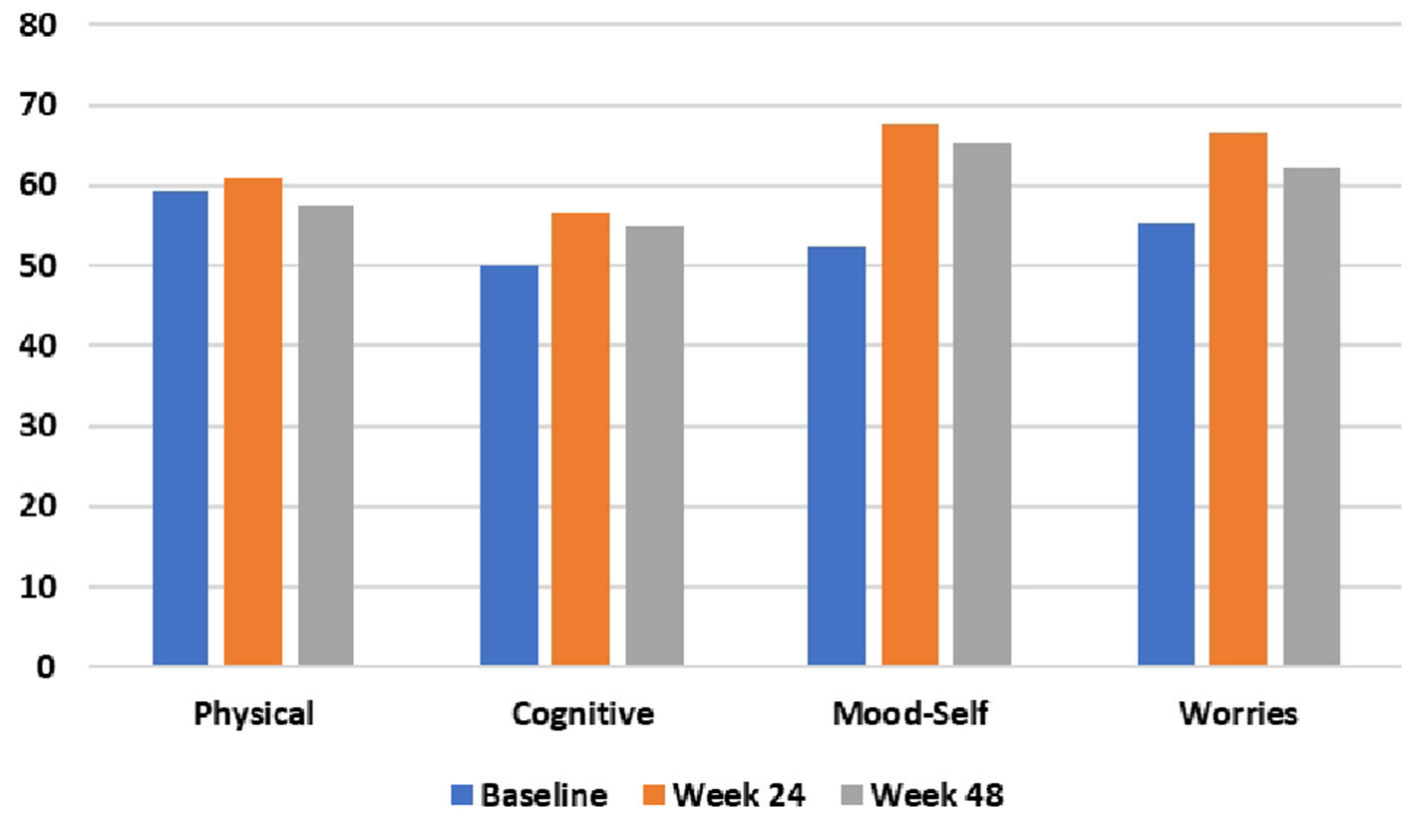
HD health-related Quality of Life (HDQoL) outcomes at baseline, 24 weeks, and 48 weeks after commencing the time-restricted ketogenic diet (higher numbers indicate improved quality of life).

During the 48-week TRKD, our patient's mean blood glucose and BHB levels (±standard deviation) were measured at 9.71 ± 2.48 and 0.90 ± 0.57 mmol/L, respectively. During this time, his diabetes specialist altered the insulin glusiline to 5 units bd and insulin glargine to 10.5 units daily and our patient noted a reduced frequency of hypoglycemic episodes. Improvements were measured in the TMS (which decreased from 56 to 27, representing a 52% improvement from baseline), HD-ADL (which decreased from 46 to 33, representing a 28% improvement), and cUHDRS (which increased from 8.4 to 10.1, representing a 20% improvement) (Table 1). The SDMT, Stroop, and TFC scores showed no substantial change. Improvements were also measured in the PBA-s for apathy (which decreased from 12 to 3, representing a 75% improvement), disorientation (which decreased from 8 to 0, representing a 100% improvement), anger (which decreased from 6 to 3, representing a 50% improvement), and irritability (which decreased from 6 to 2, representing a 67% improvement) (Figure 2). The only worsened behavior problem was increased anxiety, which arose in the setting of an unforeseen exacerbation of social stressors 8 months into the TRKD and required a brief admission to hospital followed by the addition of lorazepam 1 mg daily. With respect to quality of life, improvement was noted in mood-self (which increased from 52 to 65, representing a 25% improvement) (Figure 3). The other domains improved to a lesser degree or showed no substantial change. Our patient's weight was 62.7 kg at baseline, 62.5 kg at week 24, and 61.0 kg at week 48. With respect to blood investigations from baseline to week 48, hemoglobin and creatinine remained in the normal range, glycosylated hemoglobin (HbA1C) decreased from 63 to 61 mmol/mol, triglycerides increased from 1.7 to 2.6 mmol/L, high-density lipoprotein remained stable at 1.95 mmol/L, low-density lipoprotein increased from 3.1 to 6.0 mmol/L, and total cholesterol increased from 5.8 to 9.1 mmol/L. The only adverse effects experienced during the TRKD were decreased bowel movement frequency (from once per day to once every 2–3 days) and increased thirst, neither of which required treatment.

**Table 1:** Motor, cognitive, and functional outcomes at baseline, 24 weeks, and 48 weeks after commencing the time-restricted ketogenic diet (for the TMS and HD-ADL, lower numbers indicate improved performance, whereas for the SDMT, Stroop Tests, TFC, and cUHDRS, higher numbers indicate improved performance).

**Outcome**	**Baseline**	**Week 24**	**Week 48**
**Motor**			
TMS			
Ocular pursuit	4	0	0
Saccade initiation	2	2	2
Saccade velocity	5	2	0
Dysarthria	1	1	0
Tongue protrusion	1	1	0
Finger taps	2	4	1
Pronate/supinate—hands	4	4	2
Luria	4	0	0
Rigidity—arms	1	0	0
Bradykinesia—body	2	2	1
Maximal dystonia	13	11	7
Maximal chorea	11	6	9
Gait	1	2	1
Tandem walking	0	2	0
Retropulsion pull test	1	1	0
Diagnosis confidence level	4	3	4
Total	56	41	27
**Cognition**			
SDMT	31	32	32
Stroop tests			
Word reading (raw score)	90	93	84
Word reading (*T*-score)	42	44	37
Color (raw score)	69	67	64
Color (*T*-score)	44	43	40
Color-word (*T*-score)	62	61	56
Interference (*T*-score)	64	63	61
**Function**			
TFC			
Occupation	0	0	0
Finances	0	0	0
Domestic chores	1	1	1
Activities of daily living	3	3	3
Care level	2	2	2
Total	6	6	6
HD-ADL	46	24	33
**Composite of motor, cognition, and function**			
cUHDRS	8.4	9.6	10.1

*TMS, Total Motor Score; SDMT, Symbol Digit Modalities Test; TFC, Total Functional Capacity; HD-ADL, HD Activities of Daily Living; cUHDRS, Composite Unified HD Rating Scale*.

## Discussion

This case study is unique in that a patient with progressive, deteriorating HD was managed with a TRKD, with subsequent improvements in his motor symptoms (52% improvement from baseline), activities of daily living (28% improvement), cUHDRS score (20% improvement), most HD-related behavior problems (apathy, disorientation, anger, and irritability improved by 50–100%), and mood-related quality of life (25% improvement). Cognition did not improve. Weight remained stable and there were no significant adverse effects.

Used in isolation, fasting and ketogenic diets may each provide a bioenergetic advantage for neurons and muscle cells in a neurodegenerative disorder such as HD; however, there may be further advantages to combining them. On a practical level, time-restricted feeding eases the burden of organizing multiple meals and frees up time throughout the day, compensating for the extra time required to prepare the initially unfamiliar recipes of a ketogenic diet. In turn, the ketogenic diet improves blood insulin control and reduces long-term hunger (Ludwig et al., 2018), making the fasts more tolerable. The modified ketogenic diet used in this study additionally emphasized flexibility, palatability, and affordability whist also providing a rich variety of cuisine options, significantly alleviating the culinary and social restrictions that impede long-term success with older, conventional ketogenic diets (Cervenka et al., 2016). On a therapeutic level, since the essential aim of both fasting and ketogenic diets is to generate a series of metabolic alterations aimed at maximizing cell and mitochondria energy metabolism (Phillips, 2022), fasting probably augments the beneficial metabolic alterations induced by the ketogenic diet and vice versa.

One critical component of patient success on a metabolic strategy (particularly a ketogenic diet) is the use of blood glucose and ketone monitors to regularly check blood glucose and BHB levels, the latter being the principal ketone contributing to brain and muscle energy metabolism. Monitors are easy to operate and allow difficulties in adherence to be rapidly recognized and corrected. During the 48-week TRKD, our patient's mean blood glucose level remained relatively high at 9.71 mmol/L with only a marginal decrease in the HbA1C, indicating a modest impact on long-term glucose control. However, he also documented fewer hypoglycemic episodes during the 48-week TRKD, which likely resulted from a mitigation of the blood glucose spikes (and subsequent dips), leading to smoother control (Ludwig et al., 2018). With respect to our patient's blood ketones, the mean 48-week blood BHB level during the TRKD was 0.90 mmol/L, which is within the range of therapeutic ketosis, albeit in the lower range. Since he adhered to the recipes in the TRKD booklet, the lower ketone levels likely related to the need for daily exogenous insulin, which would have constantly suppressed ketogenesis. Our patient's underweight body-mass index may have also limited the amount of endogenous body fat available for ketogenesis during the fasting periods.

In this case study, notable improvements were measured in the motor symptoms, activities of daily living, and cUHDRS score. Pathologically, the medium spiny neurons of the striatal indirect pathway exhibit the earliest and most severe degenerative changes in HD (Reiner et al., 1988; Deng et al., 2004), leading to a relative overactivity of the direct pathway and hyperkinetic symptoms such as chorea and dystonia (Blumenstock and Dudanova, 2020). In theory, the TRKD enhanced energy metabolism in our patient's indirect medium spiny neurons, contributing to the observed 52% improvement in motor symptoms, which to our knowledge represents the first documented case of either fasting or a ketogenic diet leading to improved motor symptoms in a hyperkinetic movement disorder. By comparison, tetrabenazine, the only drug specifically licensed to treat chorea in HD, improves baseline chorea by 23.5% (Huntington Study Group, 2006) but can produce many adverse effects which limit its clinical benefit (Coppen and Roos, 2017). We also measured improvements in activities of daily living, which may be significant given that daily function is more closely linked with health-related quality of life than motor symptoms or cognition in HD (Ho et al., 2009). The 20% improvement in the cUHDRS is noteworthy as this particular measurement best characterizes the clinical progression of HD and is strongly associated with brain measures of progressive corticostriatal atrophy (Schobel et al., 2017). It is unclear why cognition did not improve at the week 48 assessment, but this finding may partly relate to a mild sedating effect of the lorazepam, which was not present at baseline or week 24.

The most marked improvements in this case study occurred with respect to behavior problems, with apathy, disorientation, anger, irritability each improving by 50–100%. From the perspective of the patient and his fiancée, the mitigation of the behavior problems represented the most beneficial aspect of the TRKD as they had undermined his social interactions and relationships, including their own. Our patient's pronounced apathy prior to commencing the TRKD was particularly concerning, as this was the factor most responsible for his preceding weight loss. Moreover, out of all the neuropsychiatric symptoms, apathy is the most consistent marker of HD progression (Tabrizi et al., 2013). Given these considerations, the observed 75% improvement in apathy is encouraging. Only one behavior problem, anxiety, worsened during the TRKD, which occurred in the setting of an unforeseen exacerbation of social stressors and was not attributed by either the patient or his fiancée to the TRKD itself.

The only adverse effects attributed to the TRKD were mild weight loss, decreased bowel movement frequency, and increased thirst. Although our patient's underweight body-mass index was a potential red flag prior to commencing the TRKD, his 1.7 kg of weight loss turned out to be considerably lower compared with the 5–6 kg of weight he lost during the preceding 12 months. This raises the possibility of a weight-sparing effect induced by the TRKD, which is consistent with the markedly delayed weight loss experienced by HD animal models on a ketogenic diet (Ruskin et al., 2011). The decreased bowel movement frequency and increased thirst were of no concern to our patient.

Since this is a case study, its major limitation is obvious and it is not possible to precisely determine the mechanism of the improvements. Beyond the hypothesis that the TRKD enhanced brain and muscle energy metabolism and mitochondria function, other possibilities include improved diabetes control, a practice effect, a medication effect, and a placebo effect. First, our patient had type 1 diabetes and experienced improved blood glucose control, which may have benefitted his energy metabolism and HD symptoms (that said, the impact on the mean blood glucose level and HbA1C was modest). Second, a practice effect might have improved our patient's performance at each subsequent assessment (this seems unlikely, as the assessments were widely spaced at 24 weeks apart). Third, beyond impacting anxiety and cognition, the institution of lorazepam 8 months into the TRKD may have contributed to our patient's improved motor symptoms and behavior problems (but it would not explain the improvements from baseline to week 24). Fourth, our patient could not be blinded to the intervention, so a placebo effect may have contributed to his improvements.

In conclusion, this case study is unique in that a patient with progressive, deteriorating HD was managed with a TRKD, with subsequent improvements in his motor symptoms, activities of daily living, cUHDRS score, most HD-related behavior problems, and mood-related quality of life. Our patient remains dedicated to his TRKD, which both he and his fiancée continue to describe as “life-changing”. Despite the limitations of this case study, the outcome is encouraging and further studies involving metabolic strategies in HD are warranted.

## Data Availability Statement

The original contributions presented in the study are included in the article/Supplementary Material, further inquiries can be directed to the corresponding author.

## Ethics Statement

Ethical review and approval was not required for the study on human participants in accordance with the local legislation and institutional requirements. The patients/participants provided their written informed consent to participate in this study. Written informed consent was obtained from the individual(s) for the publication of any potentially identifiable images or data included in this article.

## Author Contributions

MP: conception, design, coordination, interpretation, and write-up of final article. EM: conception and proof-reading of final article. MB and BR-F: coordination and proof-reading of final article. All authors contributed to the article and approved the submitted version.

## Conflict of Interest

The authors declare that the research was conducted in the absence of any commercial or financial relationships that could be construed as a potential conflict of interest.

## Publisher's Note

All claims expressed in this article are solely those of the authors and do not necessarily represent those of their affiliated organizations, or those of the publisher, the editors and the reviewers. Any product that may be evaluated in this article, or claim that may be made by its manufacturer, is not guaranteed or endorsed by the publisher.

## References

[B1] Antonini A. Leenders K. L. Spiegel R. Meier D. Vontobel P. Weigell-Weber M.. (1996). Striatal glucose metabolism and dopamine D2 receptor binding in asymptomatic gene carriers and patients with Huntington's disease. Brain. 119, 2085–2095. 10.1093/brain/119.6.2085 9010012

[B2] Blumenstock S. Dudanova I. (2020). Cortical and striatal circuits in Huntington's disease. Front. Neurosci. 14, 82. 10.3389/fnins.2020.00082 32116525 PMC7025546

[B3] Browne S. E. Bowling A. C. MacGarvey U. Baik M. J. Berger S. C. Muqit M. M.. (1997). Oxidative damage and metabolic dysfunction in Huntington's disease: selective vulnerability of the basal ganglia. Ann. Neurol. 41, 646–653. 10.1002/ana.410410514 9153527

[B4] Bylsma F. W. Rothlind J. Hall M. R. Folstein S. E. Brandt J. (1993). Assessment of adaptive functioning in Huntington's disease. Mov. Disord. 8, 183–190. 10.1002/mds.870080212 8474487

[B5] Cervenka M. C. Henry B. J. Felton E. A. Patton K. Kossoff E. H. (2016). Establishing an adult epilepsy diet center: experience, efficacy and challenges. Epilepsy Behav. 58, 61–68. 10.1016/j.yebeh.2016.02.038 27060389

[B6] Chaturvedi R. K. Adhihetty P. Shukla S. Hennessy T. Calingasan N. Yang L.. (2009). Impaired PGC-1α function in muscle in Huntington's disease. Hum. Mol. Genet. 18, 3048–3065. 10.1093/hmg/ddp243 19460884 PMC2733807

[B7] Ciammola A. Sassone J. Sciacco M. Mencacci N. E. Ripolone M. Bizzi C.. (2011). Low anaerobic threshold and increased skeletal muscle lactate production in subjects with Huntington's disease. Mov. Disord. 26, 130–137. 10.1002/mds.23258 20931633 PMC3081141

[B8] Coppen E. M. Roos R. A. C. (2017). Current pharmacological approaches to reduce chorea in Huntington's disease. Drugs. 77, 29–46. 10.1007/s40265-016-0670-4 27988871 PMC5216093

[B9] Craufurd D. Thompson J. C. Snowden J. S. (2001). Behavioral changes in Huntington disease. Neuropsychiatry Neuropsychol. Behav. Neurol. 14, 219–226. Available online at: https://journals.lww.com/cogbehavneurol/Abstract/2001/10000/Behavioral_Changes_in_Huntington_Disease.4.aspx 11725215

[B10] Cui L. Jeong H. Borovecki F. Parkhurst C. N. Tanese N. Krainc D.. (2006). Transcriptional repression of PGC-1α by mutant huntingtin leads to mitochondrial dysfunction and neurodegeneration. Cell. 127, 59–69. 10.1016/j.cell.2006.09.015 17018277

[B11] de Cabo R. Mattson M. P. (2019). Effects of intermittent fasting on health, aging, and disease. N. Engl. J. Med. 381, 2541–2551. 10.1056/NEJMra1905136 31881139

[B12] Deng Y. P. Albin R. L. Penney J. B. Young A. B. Anderson K. D. Reiner A.. (2004). Differential loss of striatal projection systems in Huntington's disease: a quantitative immunohistochemical study. J. Chem. Neuroanat. 27, 143–164. 10.1016/j.jchemneu.2004.02.005 15183201

[B13] Duan W. Guo Z. Jiang H. Ware M. Li X.-J. Mattson M. P. (2003). Dietary restriction normalizes glucose metabolism and BDNF levels, slows disease progression, and increases survival in huntingtin mutant mice. Proc. Natl. Acad. Sci. U. S. A. 100, 2911–2916. 10.1073/pnas.0536856100 12589027 PMC151440

[B14] Grafton S. T. Mazziotta J. C. Pahl J. J. St George-Hyslop P. Haines J. L. Gusella J.. (1992). Serial changes of cerebral glucose metabolism and caudate size in persons at risk for Huntington's disease. Arch. Neurol. 49, 1161–1167. 10.1001/archneur.1992.00530350075022 1444883

[B15] Gu M. Gash M. T. Mann V. M. Javoy-Agid F. Cooper J. M. Schapira A. H.. (1996). Mitochondrial defect in Huntington's disease caudate nucleus. Ann. Neurol. 39, 385–389. 10.1002/ana.410390317 8602759

[B16] Ho A. K. Gilbert A. S. Mason S. L. Goodman A. O. Barker R. A. (2009). Health-related quality of life in Huntington's disease: which factors matter most? Mov. Disord. 24, 574–578. 10.1002/mds.22412 19097181

[B17] Ho A. K. Horton M. C. Landwehrmeyer G. B. Burgunder J.-M. Tennant A. European Huntington's Disease Network. (2019). Meaningful and measurable health domains in Huntington's disease: large-scale validation of the Huntington's disease health-related quality of life questionnaire across severity stages. Value Health. 22, 712–720. 10.1016/j.jval.2019.01.016 31198189

[B18] Hungtington Study Group (1996). Unified Huntington's disease rating scale: reliability and consistency. Mov. Disord. 11, 136–142. 10.1002/mds.870110204 8684382

[B19] Huntington Study Group (2006). Tetrabenazine as antichorea therapy in Huntington disease: a randomized controlled trial. Neurology. 66, 366–372. 10.1212/01.wnl.0000198586.85250.13 16476934

[B20] Kim J. Moody J. P. Edgerly C. K. Bordiuk O. L. Cormier K. Smith K.. (2010). Mitochondrial loss, dysfunction and altered dynamics in Huntington's disease. Hum. Mol. Genet. 19, 3919–3935. 10.1093/hmg/ddq306 20660112 PMC2947400

[B21] Longo V. D. Di Tano M. Mattson M. P. Guidi N. (2021). Intermittent and periodic fasting, longevity and disease. Nat. Aging. 1, 47–59. 10.1038/s43587-020-00013-3 35310455 PMC8932957

[B22] Ludwig D. S. Willett W. C. Volek J. S. Neuhouser M. L. (2018). Dietary fat: from foe to friend? Science. 362, 764–770. 10.1126/science.aau2096 30442800

[B23] McColgan P. Tabrizi S. J. (2018). Huntington's disease: a clinical review. Eur. J. Neurol. 25, 24–34. 10.1111/ene.13413 28817209

[B24] Miller V. J. Villamena F. A. Volek J. S. (2018). Nutritional ketosis and mitohormesis: potential implications for mitochondrial function and human health. J. Nutr. Metab. 2018, 5157645. 10.1155/2018/5157645 29607218 PMC5828461

[B25] O'Callaghan C. Bertoux M. Hornberger M. (2014). Beyond and below the cortex: The contribution of striatal dysfunction to cognition and behaviour in neurodegeneration. J. Neurol. Neurosurg. Psychiatry. 85, 371–378. 10.1136/jnnp-2012-304558 23833269

[B26] Oliveira J. M. A. (2010). Nature and cause of mitochondrial dysfunction in Huntington's disease: focusing on huntingtin and the striatum. J. Neurochem. 114, 1–12. 10.1111/j.1471-4159.2010.06741.x 20403078

[B27] O'Neill B. J. (2020). Effect of low-carbohydrate diets on cardiometabolic risk, insulin resistance, and metabolic syndrome. Curr. Opin. Endocrinol. Diabetes Obes. 27, 301–307. 10.1097/MED.0000000000000569 32773574

[B28] Phillips M. C. L. (2022). Metabolic strategies in healthcare: a new era. Aging Dis. 13, 655–672. 10.14336/AD.2021.1018 35656107 PMC9116908

[B29] Reiner A. Albin R. L. Anderson K. D. D'Amato C. J. Penney J. B. Young A. B.. (1988). Differential loss of striatal projection neurons in Huntington disease. Proc. Natl. Acad. Sci. U. S. A. 85, 5733–5737. 10.1073/pnas.85.15.5733 2456581 PMC281835

[B30] Ross C. A. Tabrizi S. J. (2011). Huntington's disease: from molecular pathogenesis to clinical treatment. Lancet Neurol. 10, 83–98. 10.1016/S1474-4422(10)70245-3 21163446

[B31] Ruskin D. N. Ross J. L. Kawamura M. Ruiz T. L. Geiger J. D. Masino S. A. A.. (2011). ketogenic diet delays weight loss and does not impair working memory or motor function in the R6/2 1J mouse model of Huntington's disease. Physiol. Behav. 103, 501–507. 10.1016/j.physbeh.2011.04.001 21501628 PMC3107892

[B32] Schobel S. A. Palermo G. Auinger P. Long J. D. Ma S. Khwaja O. S.. (2017). Motor, cognitive, and functional declines contribute to a single progressive factor in early HD. Neurology. 89, 2495–2502. 10.1212/WNL.0000000000004743 29142089 PMC5729794

[B33] Stroop J. R. (1935). Studies of interference in serial verbal reactions. J. Exp. Psychol. XVIII, 643–62. 10.1037/h0054651

[B34] Tabrizi S. J. Scahill R. I. Owen G. Durr A. Leavitt B. R. Roos R. A.. (2013). Predictors of phenotypic progression and disease onset in premanifest and early-stage Huntington's disease in the TRACK-HD study: analysis of 36-month observational data. Lancet Neurol. 12, 637–649. 10.1016/S1474-4422(13)70088-7 23664844

[B35] VanItallie T. B. Nufert T. H. (2003). Ketones: metabolism's ugly duckling. Nutr. Rev. 61, 327–341. 10.1301/nr.2003.oct.327-341 14604265

[B36] Veech R. L. Chance B. Kashiwaya Y. Lardy H. A. Cahill G. F. Jr. (2001). Ketone bodies, potential therapeutic uses. IUBMB Life. 51, 241–247. 10.1080/152165401753311780 11569918

